# Low long-lasting insecticide nets (LLINs) use among household members for protection against mosquito bite in kersa, Eastern Ethiopia

**DOI:** 10.1186/1471-2458-12-914

**Published:** 2012-10-29

**Authors:** Tesfaye Gobena, Yemane Berhane, Alemayehu Worku

**Affiliations:** 1 College of Health Sciences, Haramaya University, Harar, P.O. Box: 235, Ethiopia; 2 Addis Continental Institute of Public Health, Addis Ababa, Ethiopia; 3 School of Public Health, Addis Ababa University, Addis Ababa, Ethiopia

## Abstract

**Background:**

In Ethiopia, despite the increasing availability of long-lasting insecticide treated nets (LLINs), the LLINs use among LLINs owning households has not been satisfactory. Identifying the circumstances and the associated factors is necessary to achieve the Millennium Development Goal targets. We aimed to assess barriers related with LLIN use at the household level.

**Methods:**

A cross sectional survey was conducted in Kersa Demographic Surveillance and Health Research Center (KDS-HRC) from October to November 2010. A total of 2867 households were selected from a surveillance database using a simple random sampling technique. The data were collected by interviewing women, direct observation of LLINs conditions and use, and in-depth interviewing of key informants. Multivariate analysis was used to determine independent predictors of LLIN non-use.

**Results:**

Of the total surveyed households, 65.5% (1879) had at least one LLIN, but 33.5% (630) LLINs owned households had used at least one LLIN the night before the survey. Low educational level of women, low awareness on malaria prevention, unavailability of separate sleeping room, LLIN colour preference, and unavailability of enough LLINs to the household members were the main barriers to LLIN use. A supplementary qualitative interview with key informants also identified that poor condition of LLINs; undermining the extent of malaria; and using the LLIN for other purposes as the main reasons for non-use.

**Conclusions:**

This study indicates that only about one third of LLIN owned households are actually using at least one LLIN for protection against mosquito bite. Thus, majority of the residents are at higher risk of mosquito bite and acquiring of malaria infection. Households living in fringe zone are not benefiting from the LLIN protection. Further progress in malaria prevention can be achieved by specifically targeting populations in fringe zones and conducting focused public education to increase LLIN use.

## Background

The use of insecticide-treated nets (ITNs) is one of the main malaria control strategies in most malaria endemic countries to reach the Roll Back Malaria (RBM) targets to reduce the malaria burden by 50% in 2010 compared to 2000 levels and at least 75% by 2015 [1]. Despite the large scale distribution of ITNs in many malaria endemic countries, there is a wide variation in the availability [2] and use of ITNs/LLINs at the household level [3-6].

In Ethiopia, the Ministry of Health (MOH) conducted continuously mass distribution of LLINs between 2005 and 2007, targeting to distribute two LLINs per household in malaria endemic areas [7]. In these years, households LLIN ownership increased from 3.4% to 53.3%; while use among children under-five years of age and pregnant women increased from 1.5% and 1.1% to 33.1% and 35.2%, respectively [8,9].

Empirical evidence has demonstrated the effectiveness of LLINs in sub-Saharan Africa [10,11], though actual LLIN use remains unsatisfactory [12]. There are individual, household, ITN and environmental related barriers to bednet use among ITN/LLIN owning households. Low perception of malaria resulted in the decrease of motivation of consistent mosquito net use [13]; ITN use compliance decreased during the dry season [14]. Also types of ITNs and their poor conditions [6]; beliefs and perceptions of the cause of malaria [15]; low number of ITNs [16]; and demographic characteristics [15] were barriers to mosquito net use. A recent article also mentioned that hot weather, social factors, perceived low mosquito density, technical factors such as hanging of ITNs and its inadequate availability were the major reasons to non-use of mosquito nets among net owners [17].

In order to meet the Millennium Development Goals and the Roll Back Malaria targets, it is indispensable to determine the actual levels of use and to take timely corrective actions. Thus, the objective of the study was to determine the level of LLIN use and identify barriers of their use at the household level.

## Methods

### Study setting

The study was conducted in Kersa Demographic Surveillance and Health Research Center (KDS-HRC) of Kersa district in the eastern part of Ethiopia during the high malaria transmission season, October to November 2010 [18]. The altitude of the district ranges from 1400 meters to 3200 meters above sea level. The district comprised of 35 rural and three urban kebeles (Kebele is the smallest administrative unit in Ethiopia). The district is recognized as malaria endemic and fringe zone [8,19]. According to the district health office there are malaria cases in the highland fringe localities of the district. The livelihood of most inhabitants of the rural population is mainly subsistence farming.

The KDS-HRC was established in 2007; and it conducts quarterly visit to the households under surveillance to update health and demographic events. The KDS-HRC site includes 12 randomly selected kebeles (10 rural and 2 urban kebeles) of the district. These kebeles were selected based on mix of altitudes and urban–rural composition of the district. In these kebeles, there are 48,244 residents in 10,256 households.

This study was conducted as a baseline survey for a follow up study of consistent and non consistent LLIN user households. Thus, this study reported on a cross sectional survey conducted to determine the household level of LLIN ownership and use. Once the use level was determined; households using LLINs were compared to those who did not use to identify the factors associated with LLINs non-use.

### Sample determination and sampling

A total of 1879 households, 626 LLIN users and 1253 non-users were determined based on the difference between two population proportions with the following assumptions. Based on the study of Baume *et al*.*,* 67.86% ITNs use among respondents with correct knowledge on malaria transmission and incorrect knowledge of respondents are expected to use 60.10% [6], with 95% confidence level (Zα/2) and power of the study 80%. However, ITNs ownership among households of malaria endemic areas in the country was 65.6% [9]. Thus, to obtain 1879 bednet owned households, a total of 2912 households were included in the survey. This sample size was large enough to determine both LLIN ownership and barriers of LLIN use among households.

The study sample was taken from the KDS-HRC database proportional to the size of households of each kebele using simple random sampling. The surveillance database has unique identification number for each household under surveillance. The key informants were women in LLIN owned and non-user households during the time of the survey. The informants were selected purposefully from each of the study kebele of the district using data from the survey and identification number of the households.

### Data collection methods

This survey was conducted after the main rainy season, when the population experience intense mosquito nuisance, which helped to observe and assess mosquito nets use by the residents. A total of 12 experienced and trained female data collectors, who were high school graduates and familiar with the study area; and spoke the local language, were involved. Three experienced female Diploma Nurses have continuously supervised the data collection in the field. A slightly modified Malaria Indicator Survey (MIS) questionnaire that was implemented in Ethiopia in 2007 was used for this study [20]. A qualitative component was integrated into the questionnaire to explore the reasons why some households did not use nets; and observation was used for net use and its conditions. Moreover, a total of 15 in-depth interviews conducted using a semi-structured questionnaire. The interviews’ questions and discussions focused on reasons of LLIN non-use among LLINs owners. The survey questionnaire contained questions related to socio-economic and demographic status of the households, perception and behaviors about malaria and its control methods, mosquito net ownership and use.

Questionnaire was translated into local language (Oromiffa) and pre-tested before it was administered in face-to face interviews with women of the households. The interviews were conducted during the morning hours. In addition, the data collectors were instructed to make direct observation of the LLINs to verify their condition and where it was placed in the house.

### Data analysis

The data were double entered into EpiData 3.1 by two experienced data operators. Then the data were transferred into SPSS 16.0 software. The investigators have cleaned and analysed the data using the same software program. Household ownership of LLIN was calculated as a proportion of households having at least one LLIN among the total surveyed households. LLIN use was estimated as the proportion of households using at least one LLIN in the LLIN owning households. Once the LLIN ownership and use were determined; the data were filtered into a separate file of LLIN owned households. Then households using LLINs were compared to those who did not use to identify the factors associated with LLINs non-use. Barriers of LLIN use were determined by using enter method multivariate logistic regression model. The Omnibus (P<0.05) and the Hosmer-Lomeshow (P > 0.05) tests were used to check goodness-of-fit of model. Both the open ended questions and in-depth interviews information on non-use of LLIN were coded based on the identified thematic areas.

### Ethical considerations

Ethical clearance was obtained from the Institutional Research Ethics Review Committee of the College of Health Science of Haramaya University. An informed verbal consent was obtained from each study participant prior to conducting the study. The data collectors provided advice to household members who manifested signs and symptoms of malaria during the survey to visit the nearby health facility for diagnosis and treatment.

## Results

### Study subject characteristics

A total of 2867 households and 14226 people were included in the survey. The response rate was 98.5%. Table 1 shows the proportion of selected background characteristics of the surveyed households. Of the total surveyed household members, 16.7% (2375) were children under five years of age; and among 3165 child-bearing aged women, 5.9% (188) were reported to be pregnant women (Table 2).

**Table 1:** Proportion of household by background characteristics in Kersa district, eastern Ethiopia, 2010

**Characteristics**	**Frequency (%)**
Place of residence (n=2867)	
Urban	341 (11.9)
Rural	2526(88.1)
Altitude above sea level	
≤2000 meters	535 (18.7)
>2000 meters	2332 (81.3)
HH with children < 5	
Yes	1644 (57.3)
No	1223 (42.7)
Sex of household members (n=14226)	
Male	7166 (50.4)
Female	7060 (49.6)
Household size	
1–2 persons	320 (11.2)
3–5 persons	1444 (50.4)
>= 6 persons	1103 (38.4)
Roof type	
Corrugated iron sheet	2272 (79.2)
Thatched roof and others	595 (20.8)
Number of sleeping rooms (n=2867)	
1 room	2481 (86.5)
≥ 2 rooms	386 (13.5)
IRS sprayed dwellings in last 12 months	
Yes	798 (27.8)
No	2069 (72.2)

**Table 2:** Proportion of LLINs ownership, its conditions and use among household members in Kersa district, eastern Ethiopia, 2010

	**Total Household**	**Total Population**	**Children under five**	**Pregnant women**
n	2867 (98.5%)	14226	2375 (16.7%)	188 (5.9%)
Own at least one LLIN	1879 (65.5%)	9629 (67.7%)	1647 (17.1%)	145 (4.6%)
LLIN in good Conditions	906 (48.2%)	4696 (48.8%)	851 (51.7%)	82 (56.6%)
Slept under LLIN prior night	630 (33.5%)	2453 (25.5%)	767 (46.6%)	54 (37.2%)

### LLINs ownership

Of the surveyed households, 65.5% (1879) had at least one LLIN while 34.5% (988) did not have any type of mosquito nets. The majority of LLIN owned households had either one or two LLINs irrespective of their household size. The average LLIN ownership among LLIN owned household was 1.7. Of the total 3137 reported LLINs, 62.4% (1958) LLINs were observed by the data enumerators. Only 64.2% (1258) of the total observed LLINs were in good condition, without holes that could allow finger through it. The rest 35.89% (700) LLINs were in bad conditions, and had holes that allow finger through it. The observation in the survey showed many LLINs were used for covering of household properties. In 1879 LLIN owned households, there were 9629 persons of which 17.1% (1647) were children under five and 4.6% (145) were reported to be pregnant women (Table 2). LLIN ownership was significantly higher among urban (72.1%) than rural (64.6%) households (COR=1.42, 95%CI=1.20-1.82). LLIN ownership was also proportionally higher in malaria endemic zone (89.2%) and a house with corrugated iron sheet roof (68.2%) than fringe zone (60.1%) and thatched roof houses (55.5%), respectively (Table 3).

**Table 3:** Factors associated with ownership of at least one LLIN among households, Kersa district, eastern Ethiopia, 2010

**Variables**	**Ownership of at least 1 LLIN**		**Crude OR(95%CI)**	**P value**
	**Yes**	**No**		
	**No. (%)**	**No. (%)**		
Place of residence				
Urban	246 (13.1)	95 (9.6)	1.42 (1.10,1.82)	0.006
Rural	1633 (86.9)	893 (90.4)	1	
Altitude above sea level				
≤ 2000 meters	477 (25.4)	58 (5.9)	5.45 (4.10,7.25)	0.001
> 2000 meters	1402 (74.6)	930 (94.1)	1	
HH with under 5 children				
Yes	1099 (58.5)	545 (55.2)	1.15 (0.98,1.34)	0.087
No	780 (41.5)	443 (44.8)	1	
Household size				
1–2 persons	179 (9.5)	141 (14.3)	0.52 (0.41,0.68)	0.001
3–5 persons	919 (48.9)	525 (53.1)	0.72 (0.61,0.85)	0.001
≥ 6 persons	781 (41.6)	322 (32.6)	1	
Roof types				
CIS	1549 (82.4)	723 (73.2)	1.72 (1.43,2.07)	0.001
TR and others	330 (17.6)	265 (26.8)	1	
Number of sleeping rooms				
1 room	1562 (83.1)	919 (93.0)	0.37 (0.28,0.49)	0.001
≥ 2 rooms	317 (16.9)	69 (7.0)	1	
Radio possession				
Yes	692 (36.8)	454 (45.9)	0.69 (0.59,0.80)	0.001
No	1187 (63.2)	534 (54.1)	1	
IRS sprayed in last 12 months				
Yes	662 (35.2)	136 (13.8)	3.41 (2.78,4.18)	0.001
No	1217 (64.8)	852 (86.2)	1	
Malaria preventable				
Yes	1296 (69.0)	658 (66.6)	0.72 (0.58,0.89)	0.002
No	193 (10.3)	188 (19.0)	0.37 (0.28,0.49)	0.001
I don’t know	390 (20.8)	142 (14.4)	1	
Educational status of household head				
Illiterate	1397 (74.3)	718 (72.7)	0.93 (0.73,1.18)	0.567
1–4 grades	240 (12.8)	154 (15.6)	0.75 (0.55,1.01)	0.057
≥ 5 grade	242 (12.9)	116 (11.7)	1	
Educational status of household women				
Illiterate	1642 (87.4)	844 (85.4)	0.90 (0.66,1.23)	0.511
1–4 grades	101 (5.4)	81 (8.2)	0.58 (0.38,0.88)	0.010
≥ 5 grade	136 (7.2)	63 (6.4)	1	
Highest educational status of household members				
Illiterate	639 (34.0)	347 (35.1)	0.97 (0.81,1.17)	0.770
1–4 grades	570 (30.3)	287 (29.0)	1.05 (0.87,1.27)	0.622
≥ 5 grade	670 (35.7)	354 (35.9)	1	

Note: CIS, corrugated iron sheet; HH, household; IRS, indoor residual spraying; n, study subjects; TR, thatched roof.

### Utilization of LLINs

In 33.5% (630) of the households, at least one LLIN was used in previous night of the survey and 66.5% (1249) did not use. Of these, 62.2% (392) of the households hanged at least one of their LLIN above bed/sleeping place in the room. Of 9629 persons in the LLIN owned households, only 25.5% (2453) household members, including 46.6% (767) of children under five and 37.2% (54) pregnant women slept under LLIN. The binary logistic regression analysis indicated that urban resident households (COR=0.73, 95%CI=0.54-0.98), availability of a single sleeping room (COR=0.61, 0.47-0.77), and LLIN colour preference (COR=0.42, 0.34-0.52) were less likely to use LLIN than their counterparts. However, household with children of under five (COR=1.41, 95%CI=1.16-1.72), households residing in altitude lower than 2000 meters above sea level (COR=3.47, 95%CI=2.79-4.30), household sprayed with IRS in last year (COR=4.32, 95%CI=3.52-5.30), and those with awareness on malaria prevention (COR=1.79, 95%CI=1.39-2.31) were more likely to use LLINs than their counterparts (Table 4).

**Table 4:** Factors associated with LLINs use the night prior to the survey among household members Kersa district, eastern Ethiopia, 2010

**Variables**	**LLINs use**		**COR(95%CI)**	**P value**
	**Users (%)**	**Nonusers (%)**		
Place of residence				
Urban	68 (10.8)	178 (14.3)	0.73 (0.54,0.98)	0.036
Rural	562 (89.2)	1071 (85.7)	1	
Altitude				
≤ 2000m	263 (41.7)	214 (17.1)	3.47 (2.79,4.30)	0.001
>2000m	367 (58.37)	1035 (82.9)	1	
HH with under 5 children				
Yes	403 (64.0)	697 (55.8)	1.41 (1.16,1.72)	0.001
No	227 (36.0)	552 (44.2)	1	
Household size				
1–2 persons	54 (8.6)	125 (10.0)	0.84 (0.59,1.19)	0.335
3–5 persons	311 (49.4)	608 (48.7)	0.99 (0.81,1.22)	0.969
≥ 6 persons	265 (42.1)	516 (41.3)	1	
Education of HH head				
Illiterate	448 (71.1)	949 (76.0)	0.75 (0.57,1.00)	0.053
1–4 grade	89 (14.1)	151 (12.1)	0.94 (0.65,1.37)	0.760
≥ 5grade	93 (14.8)	149 (11.9)	1	
Education of women				
Illiterate	522 (82.9)	1120 (89.7)	0.43 (0.30,0.61)	0.001
1–4 grade	37 (5.9)	64 (5.1)	0.53 (0.31,0.89)	0.018
≥ 5grade	71 (11.3)	65 (5.2)	1	
Highest education of HH member				
Illiterate	190 (30.1)	449 (35.9)	0.89 (0.70,1.13)	0.328
1–4 grade	224 (35.6)	346 (27.7)	1.36 (1.08,1.72)	0.010
≥ 5grade	216 (34.3)	454 (36.4)	1	
Sleeping rooms				
1 room	493 (78.3)	1069 (85.6)	0.61 (0.47,0.77)	0.001
≥ 2 rooms	137 (21.7)	180 (14.4)	1	
Roof types				
CIS	519 (82.4)	1030 (82.5)	0.99 (0.77,1.28)	0.964
TR and other	111 (17.6)	219 (17.5)	1	
Radio possession				
Yes	229 (36.3)	463 (37.1)	0.97 (0.79,1.18)	0.760
No	401 (63.7)	786 (62.9)	1	
IRS sprayed in last year				
Yes	363 (57.6)	299 (23.9)	4.32 (3.52,5.30)	0.001
No	267 (42.4)	950 (76.1)	1	
Malaria preventable				
Yes	491 (77.9)	805 (64.5)	1.79 (1.39,2.31)	0.001
No	40 (6.3)	153 (12.2)	0.77 (0.51,1.16)	0.215
I don’t know	99 (15.7)	291 (23.3)	1	
LLINs colour preference				
Yes	136 (21.6)	494 (39.6)	0.42 (0.34,0.52)	0.001
No	494 (78.4)	755 (60.4)	1	
Number of LLINs				
1 LLIN	223 (35.4)	565 (46.0)	0.21 (0.15,0.31)	0.001
2 LLINs	309 (49.0)	611 (49.7)	0.27 (0.19,0.39)	0.001
≥ 3 LLINs	98 (15.6)	53 (4.3)	1	

Note: CIS, corrugated iron sheet; COR, crud odds ratio; TR, Thatched roof.

While the confounder controlled the LLIN use was lower in urban (27.6%) than rural (34.4%) households (AOR=0.51, 95%CI=0.34-0.76). LLINs ownership and use were higher in malaria endemic zone (89.2%) and (55.1%) than fringe zone (60.1%) and (26.2%), respectively. Proportion of LLINs ownership was greater in households residing in a house with corrugated iron sheet roof (68.2%) than thatched roof houses (55.5%). But, there was no statistical significant effect in LLIN use while the confounders controlled (A OR=0.85, 95%CI=0.64-1.15) (Table 3 and 5).

### Barriers to LLINs use

After controlling for possible confounder using multivariate logistic regression model, barriers of LLIN use were identified among LLIN owned households. The final model showed that presence of illiterate woman in the household (AOR=0.39, 95%CI= 0.24-0.62), ITN colour preference (AOR=0.41, 95%CI=0.31-0.53), available of one LLIN (AOR=0.25, 95%CI=0.17-0.39), presence of a single sleeping room (AOR=0.60, 95%CI= 0.45-0.79), women’s lack of awareness on prevention of malaria (AOR=0.43, 95%CI= 0.27-0.68), and urban background of residents (AOR=0.51, 95%CI=0.34-0.76) were independent predictors of LLIN non-use (Table 5).

**Table 5:** Factors associated independently with LLINs use among household members, Kersa district, eastern Ethiopia 2010

**Variables**	**LLINs use**		**AOR(95%CI)**	**P value**
	**Users (%)**	**Nonusers (%)**		
Place of residence				
Urban	68 (10.8)	178 (14.3)	0.51 (0.34,0.76)	0.001
Rural	562 (89.2)	1071 (85.7)		
Altitude				
≤ 2000m	263 (41.7%)	214 (17.1)	2.56 (1.99-3.29)	0.001
>2000m	367 (58.37%)	1035 (82.9)		
HH with under 5 children				
Yes	403 (64.0%)	697 (55.8)	1.36 (1.06,1.75)	0.015
No	227 (36.0%)	552 (44.2)		
Household size				
1–2 persons	54 (8.6)	125 (10.0)	1.46 (0.91,2.35)	0.115
3–5 persons	311 (49.4)	608 (48.7)	1.27 (0.98,1.65)	0.066
≥ 6 persons	265 (42.1)	516 (41.3)		
Education of HH head				
Illiterate	448 (71.1)	949 (76.0)	0.73 (0.50,1.07)	0.104
1–4 grade	89 (14.1)	151 (12.1)	0.67 (0.43,1.05)	0.084
≥ 5grade	93 (14.8)	149 (11.9)		
Education of women				
Illiterate	522 (82.9%)	1120 (89.7)	0.39 (0.24,0.62)	0.001
1–4 grade	37 (5.9%)	64 (5.1%)	0.48 (0.26,0.89)	0.020
≥ 5grade	71 (11.3%)	65 (5.2%)		
Highest education of HH member				
Illiterate	190 (30.1)	449 (35.9)	1.16 (0.85,1.57)	0.361
1–4 grade	224 (35.6)	346 (27.7)	1.63 (1.23,2.17)	0.001
≥ 5grade	216 (34.3)	454 (36.4)		
Sleeping rooms				
1 room	493 (78.3)	1069 (85.6)	0.60 (0.45,0.74)	0.001
≥ 2 rooms	137 (21.7)	180 (14.4)		
Roof types				
CIS	519 (82.4)	1030 (82.5)	0.85 (0.64,1.15)	0.294
TR and other	111 (17.6)	219 (17.5)		
Malaria preventable				
Yes	491 (77.9)	805 (64.5)	1.09 (0.83,1.45)	0.526
No	40 (6.3)	153 (12.2)	0.43 (0.27,0.68)	0.001
I don’t know	99 (15.7)	291 (23.3)		
LLINs colour preference				
Yes	136 (21.6)	494 (39.6)	0.41 (0.31,0.53)	0.001
No	494 (78.4)	755 (60.4)		
Number of LLINs				
1 LLIN	223 (35.4)	565 (46.0)	0.25 (0.17,0.39)	0.001
2 LLINs	309 (49.0)	611 (49.7)	0.36 (0.25,0.54)	0.001
≥ 3 LLINs	98 (15.6)	53 (4.3)		

Note: AOR: adjusted odds ratio; HH, households.

The qualitative results from the open ended questions of the survey and the in-depth interviews revealed a number of reasons why they did not use LLINs. Majority of the respondents mentioned the following reasons as barriers to LLINs non-use: poor condition of the mosquito nets; non preference of the white and rectangular shape LLINs, unavailability of enough LLIN for the household members, use of fire place and sharing the same room with domestic animals, undermining the extent of malaria problem, low perception on malaria prevention using mosquito nets, and using the nets for purposes other than for the intended purpose such as for covering of household properties and using as curtain.

## Discussion

The study revealed that about two third of the surveyed households had at least one LLIN, but only one third of them used at least one of their LLIN in the previous night of the survey. This is lower than survey results in other similar studies in the country. They reported that 91.0% of the households had at least one ITN and 65% of them used at least one ITN [6]. A similar study in Wonago district, southern Ethiopia, reported 75.5% usage [16]. It was also lower than results found in other sub-Saharan countries [21-23]. But, it is almost similar with a report of other study in the country, which reported 37.0% and 19.6% of the households had used at least one mosquito net and LLIN, respectively [24]. Moreover, the study identified individual, household, socio-economic, environmental, and LLIN related characteristics as barriers to LLIN use.

In this survey, there could be a potential bias in measuring LLIN use among the entire household members of the respondent. It was found to be less likely to observe LLIN use of all the household members in the household, even if the net was available in the house and in hanged position. This may not be a problem at least to ensure use of at least one LLIN at least by one of the household members, including the young children and the respondent herself. In addition, the presence of hanged LLIN above the bed or sleeping place in the room was considered as a proxy indicator to LLIN use. The study also relied on a cross sectional survey conducted after the main rainy season when mosquito density and malaria transmission is high which LLIN use may be more likely to be higher than during the dry season. But, it may be useful for understanding of the reasons why LLIN owned households did not use it.

Though the women’s awareness on malaria prevention was moderate (68.2%), it was not translated into LLIN use in the study area, which is consistent with the other study in the country [16]. Similarly, several cross sectional studies have shown that women in some African countries have reasonably good knowledge on the cause and prevention of malaria. However, the extent of mosquito net use is not as good as their knowledge [25,26]. It may be due to the differences on the burden of mosquito bite and malaria infection and access to health information [26].

More urban households (72.1%) owned LLINs than rural (64.6%) ones (COR=1.42, 95%CI 1.10, 1.82). This is lower than Kafta-Humera district, Ethiopia where the study reported 91.1% in urban and 80.0% in rural households [27]. But higher than the 2007 MIS report, 39.5% of urban and 56.2% rural [9]. When the confounding factors are controlled, LLIN use was lower in urban (27.6%) than rural (34.4%) households (Adjusted OR=0.51; 95%CI, 0.34, 0.76). This is in contrary with Haileselassie *et al.* and the 2007 MIS survey reports, though both studies assessed ITNs use among selected household members only. There would be a couple of explanations for this difference: difference in housing construction and use, like using a separate room for cooking, presence of separate bed rooms, and expansion of health extension program in the rural part of the country could be among the reasons. The LLINs use was higher among residents of malaria endemic zone than fringe zone residents, and this is consistent with other studies in the country [6,9]. This may be attributed to low mosquito population and malaria infections in the malaria fringe zone than the malaria endemic areas. It would also be more likely that health extension workers may give more messages on malaria prevention and control methods and LLIN use to malaria endemic area residents.

Absence of separate sleeping room, sharing rooms with domestic animals and putting fire place in the room were household barriers to LLIN use. This is similar with a cross sectional study conducted in the country [16]. Sharing of one common sleeping place in the house may not be convenient to use LLIN for all of the household members. Observation also showed that most rural residents had non partitioned single room and had a fire place in the same room which may not be convenient to hang LLINs regularly. However, the sleeping habit of the community favoured the young children to share the available LLIN with their mother or father. LLIN ownership was not different between households that had children under five years (66.8%) and those did not have (63.8%). This is may be due to the universal target to LLINs coverage to all malaria risk residents. However, the presence of young children in the household and living in recognized endemic zone were independent predictors of LLIN use (Table 5).

The study also identified that LLINs colour and shape preference were barriers to bednets use. The blue and cylindrical shape LLINs were more preferable than the white and rectangular. The study also showed that the majority (91.8%) of the households had either one or two LLINs, which may be inadequate to use for big household members.

The qualitative part of the study also supported that hanging of rectangular net is inconvenient and hard to keep for a long time in hanged position in small multipurpose room. It was also mentioned that the white nets may be coated or soiled with soot and may be unsightly to use for a long time. Recent studies in Ethiopia [6,16] and in Kenya [28] have also demonstrated that ITNs size, shape, colour and the availability of ITNs in the household as the barriers to mosquito nets use. The qualitative open ended questions and the observations showed that several LLINs were in a poor condition and handled improperly. Some households had used LLINs to cover some household properties which may shorten the service years of the LLINs and may make the nets conditions poor.

## Conclusions

Although LLINs ownership was moderate, only a small proportion of the household members slept under LLINs in previous night of the survey. Thus, residents are at higher risk of mosquito bite and acquiring of malaria infection. This study also revealed the barriers of LLIN use. These barriers can be categorized under individual, household, socio-economic, LLIN and environmental related factors. Further progress in malaria prevention can be achieved by specifically targeting populations in malaria fringe zones and conducting focused public education to increase use of mosquito net.

## Competing interests

The authors declare that they have no competing interests.

## Authors’ contributions

All authors were involved in all steps from design of the research to write up of the paper. All have read and approved the paper.

## Pre-publication history

The pre-publication history for this paper can be accessed here:

http://www.biomedcentral.com/1471-2458/12/914/prepub
